# Treatment evolution for metastatic castration‐resistant prostate cancer with recent introduction of novel agents: retrospective analysis of real‐world data

**DOI:** 10.1002/cam4.576

**Published:** 2015-12-29

**Authors:** Thomas W. Flaig, Ravi C. Potluri, Yvette Ng, Mary B. Todd, Maneesha Mehra

**Affiliations:** ^1^ University of Colorado Cancer CenterMedical OncologyAuroraColorado; ^2^ Smart Analyst Inc.New YorkNew York; ^3^ Janssen Global ServicesGlobal Medical AffairsRaritanNew Jersey; ^4^ Janssen Global ServicesGlobal Market Access AnalyticsRaritanNew Jersey

**Keywords:** metastatic castration‐resistant prostate cancer, prostate cancer, real‐world, treatment patterns

## Abstract

Despite increasing drug treatment options for metastatic castration‐resistant prostate cancer (mCRPC) patients, real‐world treatment data are lacking. We conducted retrospective analyses of commercial claims and electronic medical record (EMR) databases to understand how treatment patterns for mCRPC have changed in a US‐based real‐world population. Truven Health Analytics MarketScan^®^ (2000–2013) and EMR (2004–2013) databases were used to identify patients with an index prostate cancer diagnosis (ICD‐9 codes 185X or 233.4X) and prescription claims for an mCRPC drug (mitoxantrone, estramustine, docetaxel, sipuleucel‐T, cabazitaxel, abiraterone acetate, enzalutamide, or radium‐223). Regimen analyses for first line of therapy (LOT1), second line of therapy, and beyond were performed among cohorts based on year of first mCRPC drug usage. mCRPC drug usage and treatment duration were compared across cohorts and age groups within each cohort. The commercial claims cohort yielded 3437 evaluable patients. Most men (91%) commencing mCRPC treatment had docetaxel as LOT1 in 2010; this number had declined to 15% in 2013. In 2013, 67% and 9% of patients used abiraterone acetate and enzalutamide, respectively, as LOT1. Among both commercial claims and EMR cohorts, treatment pattern changes were most pronounced in men aged >80 years, and median treatment duration for some mCRPC drugs was shorter than expected based on available clinical trial information. These results demonstrate a shift in mCRPC treatments during the past 5 years, with greater use of newer noncytotoxic treatments than docetaxel. These real‐world data aid in understanding the changing role of chemotherapy in the management of mCRPC.

## Introduction

For more than three decades, the mainstay of medical therapy for advanced prostate cancer has remained androgen deprivation therapy (ADT) via the medical disruption of the androgen‐signaling pathway 1. In men with advanced prostate cancer who started on ADT, there is a high initial response rate as measured biochemically or clinically in those with symptomatic disease 2. Ultimately, however, these men will progress despite castration levels of testosterone over time, and succumb to the disease 2, 3.

Historically, the treatment of metastatic castration‐resistant prostate cancer (mCRPC) has focused on palliation, with modest impact on patients’ survival. In addition to the mainstay of ADT, mitoxantrone was approved in 1996, based primarily on improvement of quality‐of‐life measures, as opposed to prolonged survival 4. In 2004, docetaxel was approved for mCRPC based on the results of two phase 3 trials that compared docetaxel with mitoxantrone, and showed both palliative benefit and a modest extension of survival 5, 6. Since then, docetaxel has played a major role in the treatment of mCRPC, whereas a decrease in mitoxantrone use through 2009 was reported in a population‐based study 7. However, many men, especially the elderly, were not considered candidates for cytotoxic chemotherapy and remained undertreated 8. Additionally, the responses observed with docetaxel in this setting were not durable in most men, and nearly all progressed over time 2. Clinical trials combining docetaxel with additional agents have not shown a clinically significant benefit to this combination approach 9, 10.

More recently, five new agents have been approved by the US Food and Drug Administration (FDA) for mCRPC (Table 1) 2, 11, considerably increasing treatment choices for mCRPC patients. Sipuleucel‐T is a first‐in‐class immunotherapy associated with an improvement in overall survival (OS) of men with minimally symptomatic mCRPC 12. Cabazitaxel was shown to improve OS of men with mCRPC progressing after docetaxel 13. Radium‐223 was shown to reduce pain and improve OS of men with CRPC with bone metastases and no known visceral metastases 14. There is a new understanding of the role of testosterone in men with castrate levels of systemic testosterone 11, 15, from both nongonadal sources and adaptive mechanisms in the tumor microenvironment that continue to drive prostate cancer progression via androgen biosynthesis. From this, novel hormonal agents have been investigated in mCRPC, leading to the approval of abiraterone acetate, an androgen biosynthesis inhibitor 16, and enzalutamide, an androgen receptor antagonist 17.

**Table 1:** Drugs approved for mCRPC by the US food and drug administration

Drug	Approval Date	Therapy Line and Duration Guideline^a^		MOA	Pivotal Trial
		Pre‐docetaxel^b^	Post‐docetaxel^c^		
Docetaxel	May 2004	n/a	n/a	Taxane (chemotherapy by tubulin inhibition)	TAX327 5, 6
Sipuleucel‐T	April 2010	Yes^d^	Yes^d^	Autologous cellular immunotherapy	IMPACT 12
Cabazitaxel	June 2010	No	Yes	Next generation taxane	Tropic 13
Abiraterone acetate	April 2011^e^December 2012^e^	Yes	Yes	Androgen synthesis inhibitor	COU‐AA‐301 19, 27COU‐AA‐302 28
Enzalutamide	August 2012^f^September 2014^f^	Yes	Yes	Androgen receptor antagonist	AFFIRM 20 PREVAIL 29
Radium‐223	May 2013	CRPC unfit or declined for docetaxel	Yes^g^	Bone‐directed alpha‐emitting radionuclide	ALSYMPCA 14

CRPC, castration‐resistant prostate cancer; MOA, mechanism of action; mCRPC, metastatic CRPC; *n*/a, not applicable.

a National Comprehensive Cancer Network (NCCN) Clinical Practice Guidelines in Oncology: Prostate Cancer. Version 1.2016 30.

b Asymptomatic.

c Symptomatic.

d Asymptomatic/minimally symptomatic, no visceral disease, good performance status.

e Approved in 2011 for the treatment of mCRPC post‐docetaxel; approval expanded in 2012 to chemotherapy‐naïve mCRPC.

f Approved in 2012 for the treatment of mCRPC post‐docetaxel; approval expanded in 2014 to chemotherapy‐naïve mCRPC.

g CRPC patients with symptomatic metastatic bone disease and no known visceral metastases.

With the improved toxicity profile of abiraterone acetate and enzalutamide over docetaxel, there may be a broader population of patients who are eligible for mCRPC treatment with these agents as opposed to chemotherapy. Consequently, a shift to later use of docetaxel may be anticipated. Patterns of real‐world treatment data have been infrequently reported in patients with mCRPC, with limited data on how these new agents are sequenced. We therefore conducted a retrospective analysis of commercial claims and electronic medical record (EMR) data to better understand how treatment patterns for mCRPC have changed.

## Materials and Methods

### Data source

Truven Health Analytics MarketScan^®^ Arbor, Michigan, US claims databases were used to identify the study population (hereafter called “commercial claims database”). The database contained claim‐level data of privately insured individuals. Additionally, EMR‐based data (2004–2013) compiled by IMS Health from 327 US oncology practice sites were used to identify patients for a validation cohort (hereafter called “EMR database”). All patient data were de‐identified, in compliance with the Health Insurance Portability and Accountability Act (HIPAA) regulations.

### Patient selection

Male patients aged ≥44 years were required to have a medical claim indicating an *International Classification of Diseases*, ninth revision, clinical modification (ICD‐9‐CM) diagnosis code for prostate cancer (ICD‐9 codes 185 or 233.4) occurring between 2000 and 2013. Patients were excluded if they had <180 days of eligibility history prior to first prostate cancer diagnosis date; any other diagnosis of prostate cancer within these 180 days; or other primary malignancies within 5 years prior to the index prostate cancer diagnosis.

Patients with mCRPC were defined based on exposure to ≥1 drugs approved by the FDA for mCRPC (mitoxantrone, estramustine, docetaxel, sipuleucel‐T, cabazitaxel, abiraterone acetate, enzalutamide, radium‐223). Yearly and multiyear cohorts were based on the year of first mCRPC drug usage, which was the date captured for the patient's first pharmacy claim for an mCRPC drug. We examined claims from 2000 to 2013 for three different multiyear cohorts (i.e., 2000–2003, prior to docetaxel approval; 2004–2008, docetaxel approval onward; and 2009–2013, approximate start of availability of novel agents). Within each multiyear cohort, patients were divided into three age groups: 44–64 years, 65–80 years, and >80 years. The >80 years age group cutoff was chosen to determine changes in treatment patterns for the very old, as only a small proportion of mCRPC patients aged >80 years receive cytotoxic chemotherapy currently 18.

### Regimen analysis

A regimen analysis for first line of therapy (LOT1), second line of therapy (LOT2), and beyond second line of therapy (LOT2+) was carried out among 1‐year and multiyear cohorts. Analytic rules were applied to determine the lines of treatment used for each patient: (1) Claims for an mCRPC drug without any other claim for the same drug in the 90 days prior to and 90 days after a claim were excluded. (2) The mCRPC drug treatment period started on the first date of use to the last date of use; the latter was the earliest date when there was at least a 90‐day gap before the next claim of the same drug. (3) For an oral therapy, the end date for each treatment period was the last claim date in that treatment period plus the number of days’ supply, while that for injectable therapy was the last claim date. (4) Treatment periods of each mCRPC drug were configured such that the entire treatment duration for a patient was divided into multiple intervals, each with a distinct usage of regimens (i.e., a single drug or a combination of drugs). Each successive interval was deemed a different line of treatment. (5) Detailed rules were applied to blank regimens, based on their duration and if the succeeding regimen was an expanded or reduced form of the preceding regimen. This resulted in blank regimens (no other claim of an mCRPC drug ±90 days from an mCRPC drug claim) being retained in some cases (duration exceeding 90 days, along with other conditions) and disregarded in others.

### Statistical analysis

Univariate descriptive statistics included the mean (±SD) and median values for continuous variables and relative frequencies for categorical variables. The analysis was carried out using SAS^®^ 9.3. Statistical differences were assessed with the independent sample *t*‐test and analysis of variance. Differences between cohorts regarding mCRPC drug usage were assessed by the Fisher's exact test and Pearson chi‐square test.

## Results

### Patient population

A total of 326,907 patients with an index prostate cancer diagnosis from 2000 to 2013 were identified from the commercial claims database (Table 2). The median age at the time of the index prostate cancer diagnosis was 64 years. A total of 3437 mCRPC patients with confirmed mCRPC drug usage were identified. Nearly two‐thirds of these patients were aged ≥65 years at the time of their first mCRPC drug usage, with a median age of 70 years. The first mCRPC drug usage for 62% of evaluable patients occurred between 2009 and 2013, whereas only 5% of evaluable patients were first exposed to an mCRPC drug between 2000 and 2003. From the EMR cohort, a total of 1340 mCRPC patients with confirmed mCRPC drug usage were identified (Table 3). Eighty‐three percent of these patients were aged ≥65 years at the time of their first mCRPC drug usage, with a median age 4 years older than patients in the 2009–2013 commercial claims cohort. The first mCRPC drug usage for 99% of evaluable patients from the EMR cohort occurred between 2009 and 2013. This observation likely reflects the increased use of EMRs after 2011; thus, the EMR data between these years was the focus of the validation cohort analysis.

**Table 2:** Distribution of mCRPC cohorts based on age at time of first mCRPC drug usage

	Commercial Claims Database				
	Total prostate cancer Population^a^	Total evaluable mCRPC Patients^b^	mCRPC Cohort^b^^,^ c		
			2000–2003	2004–2008	2009–2013
*n*	326,907^d^	3437^e^	175	1124	2138
Age, years					
Median (interquartile range)	64.0 (59–74)	70.0 (62–78)	69.0 (61–77)	71.0 (61–78)	70.0 (62–78)
Age group, *n* (%)					
44–64 years	164,499 (50.3)	1215 (35)	65 (37)	391 (35)	759 (36)
65–80 years	123,658 (37.8)	1604 (47)	86 (49)	547 (49)	971 (45)
>80 years	33,093 (10.1)	604 (18)	22 (13)	180 (16)	402 (19)

mCRPC, metastatic castration‐resistant prostate cancer.

a Age at time of first prostate cancer diagnosis.

b Based on age at time of first mCRPC drug usage.

c 14 patients were not included in the age group splits due to inconsistency or missing values in their date‐of‐birth data.

d Data for 5657 patients were not included due to inconsistency in date‐of‐birth data.

e 227 patients who had only 1 mCRPC injectable claim in the database were not deemed evaluable.

**Table 3:** Distribution of 2009–2013 mCRPC commercial claims and EMR cohorts based on age at time of first mCRPC drug usage

	2009–2013 mCRPC Cohort	
	Commercial claims*n* = 2138^a^	EMR*n* = 1340
Age, years		
Median (interquartile range)	70.0 (62–78)	74.0 (67–80)
Age group, *n* (%)		
44–64 years	759 (35.5)	233 (17.4)
65–80 years	971 (45.4)	776 (57.9)
>80 years	402 (18.8)	331 (24.7)

EMR, electronic medical record; mCRPC, metastatic castration‐resistant prostate cancer.

a 14 patients were not included in the age group splits due to inconsistency or missing values in their date‐of‐birth data.

Preferred provider organization and comprehensive plan types were the most common insurance plans among the commercial claims mCRPC population (Table S1). The majority of patients had health insurance through a preferred provider and/or a health services network plan.

### Treatment patterns from 2000 to 2013 for patients with mCRPC

The proportion of cytotoxic chemotherapy and nonchemotherapy usage among the 2000–2003, 2004–2008, and 2009–2013 cohorts was significantly different (*P* < 0.0001 for both LOT1 and LOT2). For the 2000–2003 commercial claims cohort, a total of 175 and 66 patients used an mCRPC drug for LOT1 and LOT2+, respectively (Table 4). In the 2009–2013 commercial claims cohort, 2138 and 789 patients used a mCRPC drug for LOT1 and LOT2, respectively, and 918 patients used a nonchemotherapy agent for LOT1. There was no observed usage of a nonchemotherapy regimen (abiraterone acetate, enzalutamide, sipuleucel‐T) for LOT1 among the 2000–2003 and 2004–2008 cohorts (these newer agents were not approved in the periods of 2000–2003 and 2004–2008).

**Table 4:** mCRPC drug usage by 4 index year–based cohort

	Commercial Claims Database					
	Number of patients using an mCRPC drug regimen^a^		Number of chemotherapy^b^ vsnonchemotherapy^c^ mCRPC drug regimens^d^			
Cohort	LOT1	LOT2+	LOT1 chemotherapy	LOT1 Nonchemotherapy agent	LOT2+ chemotherapy	LOT2+ nonchemotherapy agent
2000–2003	175	66	166	0	65	0
2004–2008	1124	362	1077	0	314	33
2009–2013	2138	789	1149	918	344	586

LOT1, first line of therapy; LOT2+, beyond second line of therapy; mCRPC, metastatic castration‐resistant prostate cancer.

a Excludes patients who used carboplatin.

b Cabazitaxel, docetaxel, estramustine, mitoxantrone.

c Abiraterone acetate, enzalutamide, sipuleucel‐T.

d Differences in utilization pattern of chemotherapy and nonchemotherapy mCRPC drug regimens were significant in LOT1 (*P* < 0.0001) and LOT2+ (*P* < 0.0001).

**Figure 1:**
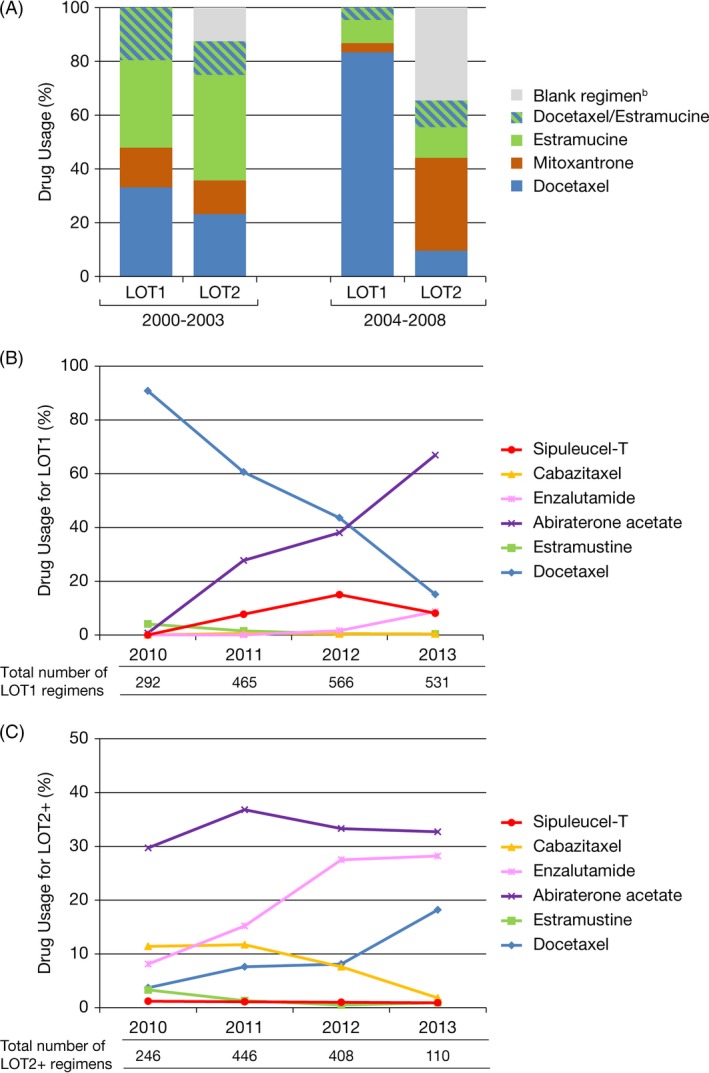
Metastatic castration‐resistant prostate cancer (mCRPC) drug usage proportion. (A) 2000–2003 and 2004–2008 commercial claims cohorts^a^; (B) 1‐year cohorts from 2010 to 2013 for LOT1; (C) 1‐year cohorts from 2010 to 2013 for LOT2+. LOT1, first line of treatment; LOT2, second line of treatment; LOT2+, beyond second line of treatment. ^a^In each of LOT1 and LOT2 settings, mCRPC drug usage proportion for each of these agents was significantly different (*P* < 0.0001) comparing the 2000–2003 and 2004–2008 commercial claims cohorts. ^b^No other claim of an mCRPC drug ±90 days from an mCRPC drug claim.

Docetaxel comprised the predominant share of mCRPC drug regimens between 2000 and 2008 (Fig. 1A, Table S2). For the 2000–2003 commercial claims cohort, 33% and 32% of patients used docetaxel and estramustine, respectively, as LOT1. More than one‐third (34%) of patients in the 2004–2008 cohort did not use a mCRPC drug for LOT2.

Noncytotoxic mCRPC drugs emerged as prominent regimens for the 2009–2013 commercial claims and EMR cohorts, corresponding to their approvals for the mCRPC treatment in 2010–2012. In the 2010 commercial claims cohort, 91% of patients used docetaxel as LOT1; by 2013, docetaxel usage decreased to 15% of LOT1 mCRPC drug regimens (Fig. 1B and C, Table S3). In the 2013 commercial claims cohort, 67%, 9%, <1%, and 8% of patients used abiraterone acetate, enzalutamide, cabazitaxel, and sipuleucel‐T, respectively, as LOT1. Similar treatment patterns were observed in the 2012 and 2013 EMR cohorts (Fig. S1). In the 2013 EMR cohort, docetaxel was used by 13% of patients as LOT1, whereas 66%, 10%, 0%, and 6.5% of patients used abiraterone acetate, enzalutamide, cabazitaxel, and sipuleucel‐T, respectively. For LOT2+, the highest proportion of mCRPC drug regimens in both the 2013 commercial claims and EMR 2013 cohorts was for abiraterone acetate, followed by enzalutamide and docetaxel (Fig. 1C, Fig. S1).

**Figure 2:**
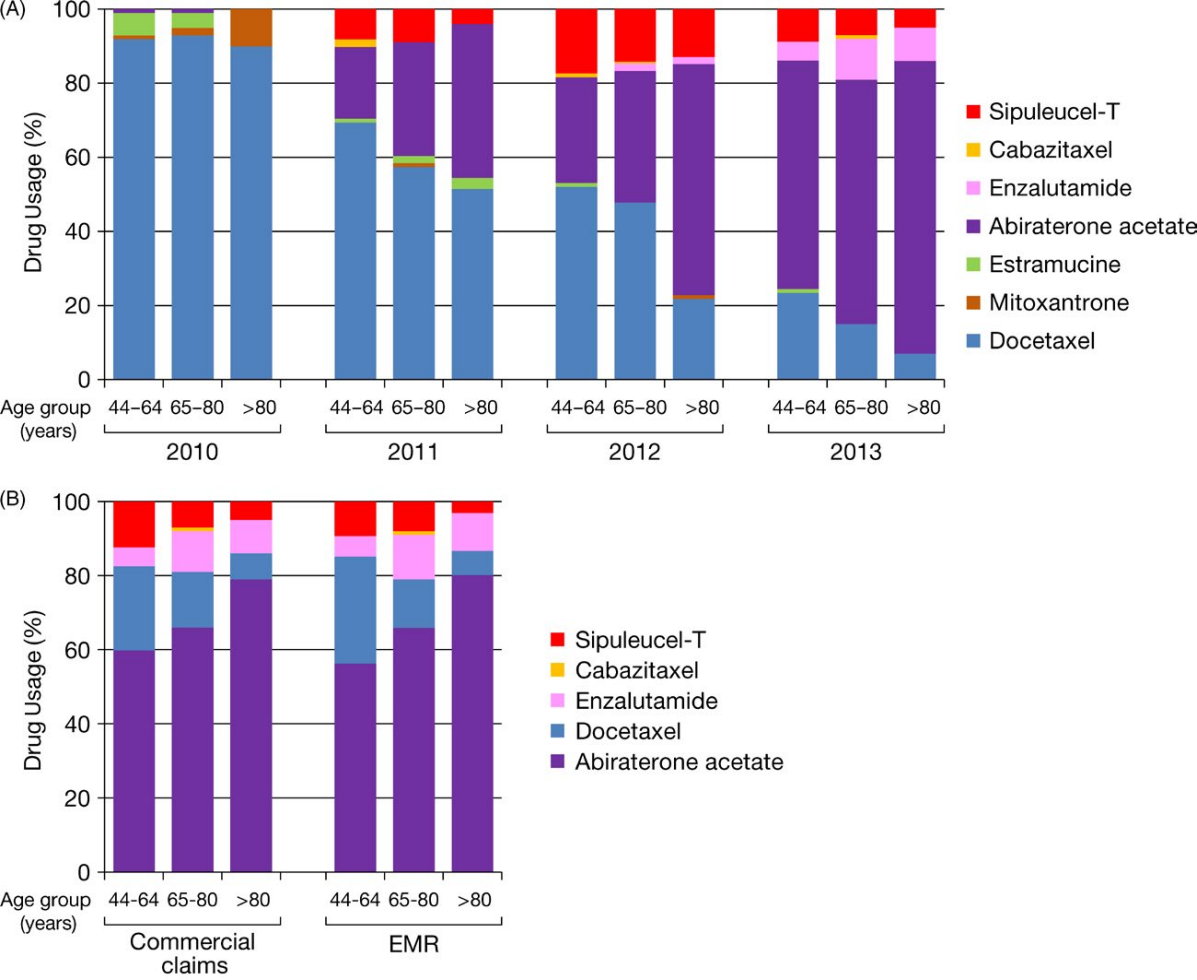
Metastatic castration‐resistant prostate cancer (mCRPC) usage proportion for LOT1 by age group. (A) individual year commercial claims cohorts from 2010 to 2013^a^; (B) 2013 commercial claims and EMR cohorts. EMR, electronic medical record; LOT1, first line of treatment. ^a^mCRPC drug usage proportion in each of the age groups (44–64, 65–80, >80 years) was significantly different (*P* < 0.0001) for each of these agents comparing years 2010–2013.

Changes in the proportion of mCRPC drug usage across annual cohorts were significantly associated with each age subgroup (all *P* < 0.0001). Among the 2010 commercial claims cohort, docetaxel usage was consistently at around 90% in all age subgroups (Fig. 2A, Table S4). In 2013, docetaxel usage was reduced substantially; there was also a difference in its usage across the age subgroups (22%, 15%, and 7% of patients in the 44–64, 65–80, and >80 years groups, respectively). This decline in chemotherapy usage was counterpoised by an increase in the usage of noncytotoxic agents, with abiraterone acetate emerging as the dominant agent. A similar pattern was observed among the 2013 EMR cohort. Among patients aged >80 years, abiraterone acetate was used in LOT1 by 79% and 78% of patients from the 2013 commercial claims and EMR cohorts, respectively (Fig. 2B, Table S5). Enzalutamide and sipuleucel‐T accounted for 9% and 5% versus 10% and 3% of LOT1 among patients aged >80 years in the 2013 commercial claims versus EMR cohorts, respectively. The changes in the proportion of mCRPC drug usage across annual cohorts were not significantly different between the commercial claims and EMR datasets for the three age subgroups.

### Treatment duration of mCRPC drugs and usage by prior chemotherapy

**Figure 3:**
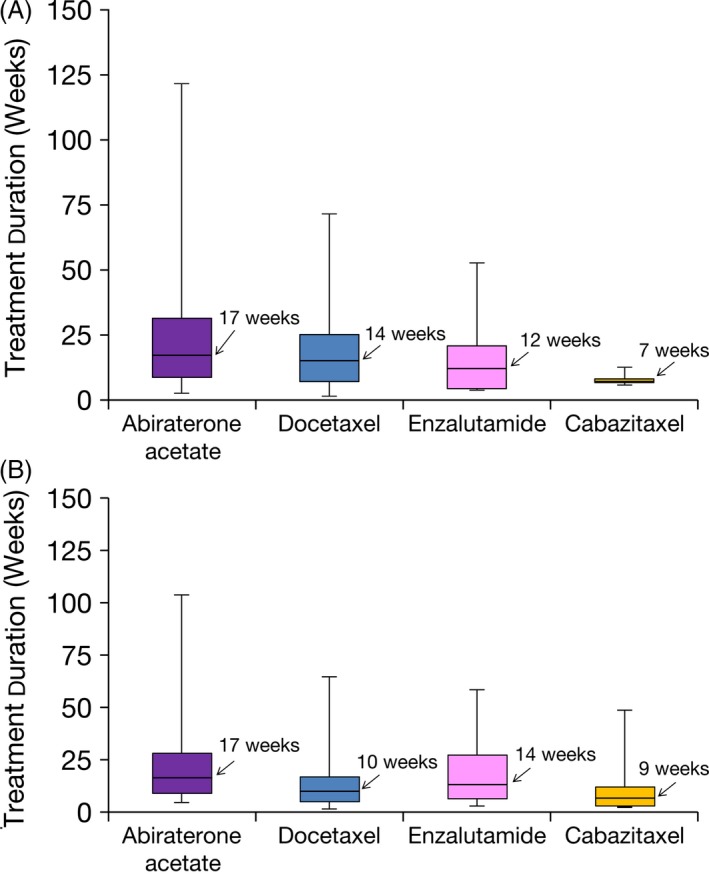
Estimated median treatment duration of metastatic castration‐resistant prostate cancer (mCRPC) drugs^a^ in the 2000–2013 commercial claims cohort. (A) LOT1; (B) LOT2. LOT1, first line of treatment; LOT2, second line of treatment. Horizontal line = median; box = 25% and 75% quartiles, whisker = minimum and maximum values. ^a^Sipuleucel‐T was not included because of the fixed‐duration treatment course.

In the commercial claims cohort, the longest median treatment durations for LOT1 were observed for abiraterone acetate (122 days) and docetaxel (100 days) (Fig. 3, Table S6); the shortest median treatment duration was observed with cabazitaxel (49 days). For LOT2, abiraterone acetate and enzalutamide had the longest median treatment duration, followed by docetaxel and cabazitaxel. The shortest median treatment duration for LOT2 was observed with estramustine (41 days).

**Figure 4:**
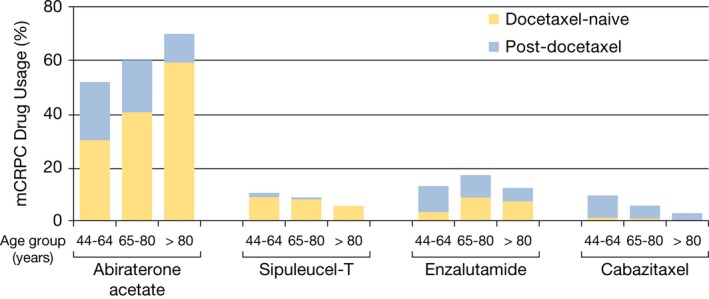
Metastatic castration‐resistant prostate cancer (mCRPC) drug usage proportion among docetaxel‐naïve and post‐docetaxel patients by age group in the 2010–2013 commercial claims cohort. In each of the age groups (44–64, 65–80, >80 years), the drug usage proportion of abiraterone acetate (*P* < 0.0001) and enzalutamide (*P* = 0.0003) was significantly different between the docetaxel‐naïve and post‐docetaxel settings, whereas the drug usage proportion of sipuleucel‐T (*P *= 0.40) and cabazitaxel (*P* = 0.42) was not significantly different between the docetaxel‐naïve and post‐docetaxel settings.

Among patients aged 65–80 years and >80 years, the use of noncytotoxic agents was more prominent among those who were docetaxel‐naïve and there was an increased proportion of abiraterone acetate and enzalutamide usage among docetaxel‐naïve patients with increasing age (Fig. 4). The utilization patterns of abiraterone acetate and enzalutamide were significantly higher in patients in the docetaxel‐naïve, versus post‐docetaxel setting (*P* < 0.0001 and *P* = 0.0003, respectively). The use of cabazitaxel was less prominent in the docetaxel‐naïve, setting, in which there was a decreased proportion of prescription claims for cabazitaxel among patients with increasing age.

## Discussion

This retrospective analysis demonstrates that there has been a considerable shift in the treatment patterns of mCRPC since 2010 in accordance with the approval of five new medical therapies for its treatment, which have dramatically increased the treatment options for this patient population.

The use of cytotoxic drugs—docetaxel in LOT1 and cabazitaxel in LOT2—has seen a remarkable decline. In contrast, the use of highly effective antiandrogen therapy (HEAT)—abiraterone acetate and enzalutamide—has become the dominant approach in both LOT1 and LOT2 mCRPC settings. Additionally, the median duration of therapy is generally longer with HEAT compared with the cytotoxic agents used for mCRPC, suggesting a net gain in clinical benefit and tolerance to patients who receive it. With the shift to new agents and more options in general, there are several emerging questions about the future treatment of mCRPC: Should treatment start earlier in the course of mCRPC, a disease state that frequently lasts several years? Will more patients be eligible for treatment with better‐tolerated agents? What is the best sequence of therapies? Will combination therapy with new agents be more beneficial than the current sequential therapy?

Changes in the treatment patterns in LOT1 were among the most dramatic. Prior to 2010, approved treatment options for mCRPC were limited and first‐line docetaxel was common. From 2010 to 2013, the proportion of men in the commercial claims cohort who used for docetaxel as LOT1 decreased from 91% to 15%, with LOT2 usage increasing modestly during this period, from 4% to 18%, suggesting a decrease and delay in docetaxel use for mCRPC. While the magnitude of these changes is notable, the overall shift from more toxic intravenous therapy to less toxic oral therapy itself is not surprising. During the observation period of this analysis, sipuleucel‐T and radium‐223 were approved for use in the pre‐docetaxel period; abiraterone acetate in the pre‐ and post‐docetaxel settings. This observation is reflective of the changes noted with the use of enzalutamide and cabazitaxel in the post‐docetaxel setting, consistent with their labeling at the time of this data collection.

Usage of HEAT as LOT2+ was observed in the 2010 cohort. It should be noted that the yearly cohorts were defined on the basis of the first mCRPC drug usage. Thus, while the 2010 cohort would have had their LOT1 usage in 2010, their LOT2+ usage would have been more likely in 2011 and beyond, during the years when most of the newer agents were approved by the FDA for mCRPC. In addition, a small proportion of the commercial claims data may reflect mCRPC treatment exposure as part of a clinical trial and early access program.

The duration of therapy data (Fig. 3) provide additional insights into the use of the new medications for mCRPC. There is a general pattern of longer treatment duration, and presumed clinical benefit and tolerance, for HEAT as opposed to cytotoxic therapy. Notably, the treatment duration decreased for docetaxel in later LOTs, while the duration increased for HEAT with each LOT. For docetaxel, this is likely due to poorer tolerance of cytotoxic therapy later in the disease course. In contrast, the longer treatment duration for HEAT may reflect the good tolerability of these drugs, the observation of clinical benefit, and a paucity of other well‐tolerated alternatives in that clinical setting. For LOT1, the median duration of abiraterone acetate and enzalutamide usage was 122 and 87 days, respectively, in the commercial claims database. This treatment duration was shorter than that expected based on the pivotal phase 3 trials for these drugs 19, 20, 21. This could reflect either real‐world use in a broader range of patients than were included in clinical trials, or heavier reliance on prostate‐specific antigen (PSA) increases triggering discontinuation of therapy, which was discouraged in the clinical trials. Further investigation into the reason for drug discontinuation is needed to ensure maximal benefit is being achieved in real‐world use.

Treatment decisions are important in older patients with mCRPC, considering the higher burden of medical comorbidities and physical limitations in this population. In the 2009–2013 cohort, the majority of patients (67%) were aged >80 years, and we observed distinct changes in treatment patterns across younger and older age cohorts. A shift away from treatment with docetaxel from 2010–2013 was most pronounced in men aged >80 years. Along these same lines, an assessment of the absolute use of any mCRPC drug in men aged >80 years showed that there were 132 total mCRPC regimens observed in 2013, compared with 41 total mCRPC regimens in 2010, suggesting an expanding group of eligible patients for this less toxic therapy.

Historically, there have been concerns that elderly patients have been underrepresented in prostate cancer clinical trials and that little is known about the real‐world treatment of this age group 22. In a modern population‐based study in Sweden of 2677 men with CRPC, 61% of men aged <70 years received chemotherapy compared with 5% of men aged >80 years, emphasizing the low utilization of chemotherapy in older mCRPC patients 18. More recently, in phase 3 trials of HEAT, elderly patients with mCRPC following chemotherapy were well represented. Treatment with enzalutamide 20 and abiraterone acetate 20 prolonged survival in men aged ≥65 years with mCRPC after docetaxel. Additionally, men ≥75 years with mCRPC treated with abiraterone acetate had improved OS, time to PSA progression, radiographic progression‐free survival, and PSA responses compared with placebo 23. Our results suggest that there has been a shift to the use of nonchemotherapy mCRPC drugs among older men in the real‐world setting. Compared with younger patients, elderly men are more likely to present with advanced disease 24, and elderly men will continue to represent an increasing proportion of the mCRPC population needing access to nonchemotherapy mCRPC treatments.

Limitations of this retrospective analysis include the limited follow‐up of patients, particularly in the 2010–2013 cohort, and the ICD‐9 coding variations across practice types and geographic regions. In addition, the database represents privately insured individuals whose socioeconomic status may differ from that of the general population. Acknowledging these limitations, some internal crosschecking provides reassurance of data fidelity. The median duration of sipuleucel‐T administration (28 days) is consistent with the approved dosing regimen of three doses every 2 weeks. Moreover, the marked change in the use of docetaxel and mitoxantrone following the approval of docetaxel for advanced prostate cancer in 2004 fits expectations and historical trends. Another strength of this analysis is the utilization of both commercial claims and EMR databases, which are separate, and independent data sources to assess mCRPC treatment patterns for the period of dramatic change.

These real‐world data provide a greater understanding of contemporary treatment of mCRPC beyond that discernable from clinical trial populations. Although randomized clinical trials are the gold standard by which to demonstrate the efficacy of new drug treatments, the study designs used in clinical trials typically place limitations on provider actions 25, and patients are often selected based on strict inclusion and exclusion criteria to enroll a defined, homogenous population, which can limit the generalizability of results to real‐world clinical practice settings 26.

Treatment options for mCRPC have expanded in recent years with the approval of five new agents. There has been a dramatic shift in the treatment of mCRPC patients in LOT1 and LOT2, from cytotoxic chemotherapy (docetaxel) to HEAT. The shift away from cytotoxic therapy was most pronounced in elderly patients, mostly likely due to the better tolerance of HEAT, and this holds the potential of expanding the proportion of men able to receive treatment for mCRPC. Many questions remain, including the optimal timing, sequencing, and potential combination of these new agents. An understanding of the current treatment patterns will be essential to designing the next generation of clinical trials for mCRPC.

## Conflict of Interest

Thomas W. Flaig has served as consultant/advisor to GTX and Janssen and received research funding from BN ImmunoTherapeutics, Cougar Biotechnology, Dendreon, Genentech, GTx, Janssen Oncology, Medivation, Novartis, Sanofi, and Exelexis. Ravi C. Potluri has served as a consultant/advisor to Janssen. Maneesha Mehra, Mary B. Todd, and Yvette Ng are employees of Janssen Research & Development and hold stock in Johnson & Johnson.

## Supporting information


**Figure S1**. mCRPC drug usage proportion among the 2012 and 2013 commercial claims and EMR cohorts.

**Table S1.** Insurance plan types in the mCRPC population: commercial claims database

**Table S2.** mCRPC drug usage proportion among the 2000–2003 and 2004–2008 cohorts.

**Table S3.** mCRPC drug usage proportion by 1‐year cohorts from 2010 to 2013 for LOT1 and LOT2+.

**Table S4.** mCRPC Drug Usage Proportion for LOT1 by Age Group and 1‐Year Cohorts From 2010–2013.

**Table S5.** mCRPC drug usage proportion for LOT1 by age group from the 2013 commercial claims and EMR Cohorts^a^.

**Table S6.** Estimated treatment duration (Days) of mCRPC Drugs^a^ in the 2009–2013 Cohorts.
