# Supplementary figures and images for: Treatment evolution for metastatic castration‐resistant prostate cancer with recent introduction of novel agents: retrospective analysis of real‐world data
Source: Cancer Med. 2015 Dec 29;5(2):182–91. doi: 10.1002/cam4.576 (PMC4735776; doi:10.1002/cam4.576)

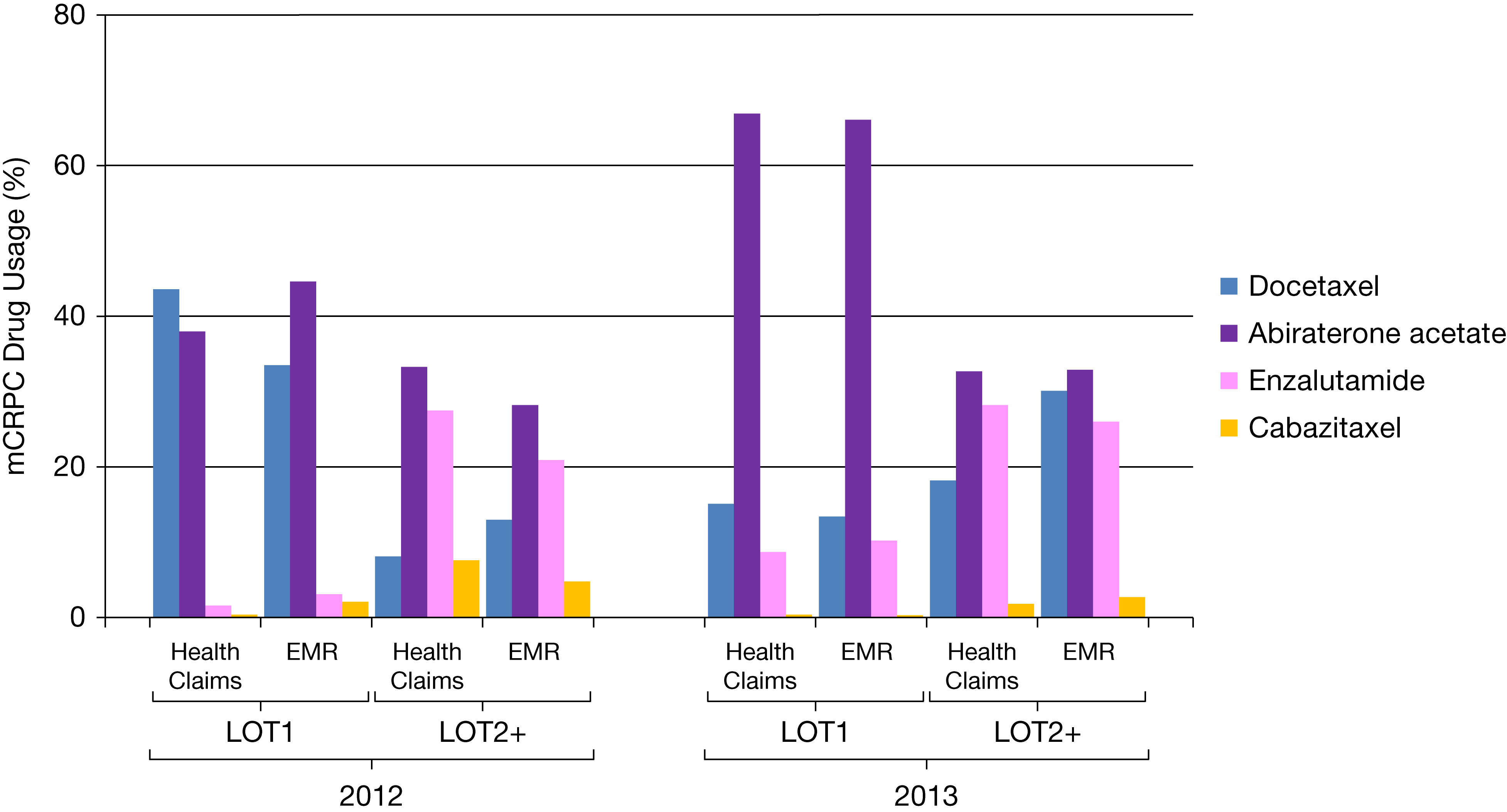

Supplement: Supplementary file 1 — Figure S1. mCRPC drug usage proportion among the 2012 and 2013 commercial claims and EMR cohorts. [file CAM4-5-182-s001.tif]
