# Supplementary material for: Treatment evolution for metastatic castration‐resistant prostate cancer with recent introduction of novel agents: retrospective analysis of real‐world data
Source: Cancer Med. 2015 Dec 29;5(2):182–91. doi: 10.1002/cam4.576 (PMC4735776; doi:10.1002/cam4.576)
Supplement: Supplementary file 2 — Table S1. Insurance plan types in the mCRPC population: commercial claims database [file CAM4-5-182-s002.docx]

**SUPPLEMENTARY TABLE 1.** Insurance Plan Types in the mCRPC Population: Commercial Claims Database

| Plan Type^a^ | mCRPC Population  N = 3437 | |
| --- | --- | --- |
|  | n | Patients (%)^b^ |
| Preferred provider organization (PPO) | 1299 | 37.8 |
| Comprehensive (COMP) | 1258 | 36.6 |
| Health maintenance organization (HMO) | 311 | 9.0 |
| Noncapitated point of service (Non-Cap POS) | 160 | 4.7 |
| Capitated or partially capitated point of service (Cap or Part Cap POS) | 54 | 1.6 |
| Consumer-driven health plan (CDHP) | 46 | 1.3 |
| High-deductible health plan (HDHP) | 12 | 0.3 |
| Exclusive provider organization (EPO) | 9 | 0.3 |

Abbreviation: mCRPC, metastatic castration-resistant prostate cancer.

^a^ Insurance plan type data were not available for all patients. Patients who show more than 1 type in their claim history have been counted in each respective plan type for the above analysis. Data includes Medicare patients with supplemental insurance.

^b^ Calculated as number of patients on a plan type as a percentage of the 3437 total patients using a mCRPC drug regimen.
