# Supplementary material for: Treatment evolution for metastatic castration‐resistant prostate cancer with recent introduction of novel agents: retrospective analysis of real‐world data
Source: Cancer Med. 2015 Dec 29;5(2):182–91. doi: 10.1002/cam4.576 (PMC4735776; doi:10.1002/cam4.576)
Supplement: Supplementary file 3 — Table S2. mCRPC drug usage proportion among the 2000–2003 and 2004–2008 cohorts. [file CAM4-5-182-s003.docx]

**SUPPLEMENTARY TABLE 2.** mCRPC Drug Usage Proportion Among the 2000 to 2003 and 2004 to 2008 Cohorts

|  | **Commercial Claims Database** | | | |
| --- | --- | --- | --- | --- |
|  | **2000-2003** | | **2004-2008** | |
| **mCRPC drug (%)** | **LOT1** | **LOT2** | **LOT1** | **LOT2** |
| Docetaxel | 32.6 | 22.8 | 83.4 | 9.4 |
| Mitoxantrone | 14.5 | 12.3 | 3.4 | 33.9 |
| Estramustine | 32.0 | 38.6 | 8.6 | 11.2 |
| Docetaxel, estramustine | 19.2 | 12.3 | 4.6 | 9.7 |
| Blank regimen | n/a | 12.3 | n/a | 33.9 |
| Abiraterone acetate | n/a | | n/a | 1.1 |
| Total number of regimens | 172 | 57 | 1113 | 277 |

Abbreviations: LOT1, first line of treatment; LOT2, second line of treatment; mCRPC, metastatic castration-resistant prostate cancer. n/a, not applicable.
