# Supplementary material for: Treatment evolution for metastatic castration‐resistant prostate cancer with recent introduction of novel agents: retrospective analysis of real‐world data
Source: Cancer Med. 2015 Dec 29;5(2):182–91. doi: 10.1002/cam4.576 (PMC4735776; doi:10.1002/cam4.576)
Supplement: Supplementary file 4 — Table S3. mCRPC drug usage proportion by 1‐year cohorts from 2010 to 2013 for LOT1 and LOT2+. [file CAM4-5-182-s004.docx]

**SUPPLEMENTARY TABLE 3.** mCRPC Drug Usage Proportion by 1-Year Cohorts From 2010 to 2013 for LOT1 and LOT2+

|  | **Commercial Claims Database** | | | |
| --- | --- | --- | --- | --- |
|  | **2010** | **2011** | **2012** | **2013** |
| **LOT1 mCRPC drug (%)** |  |  |  |  |
| Docetaxel | 90.8 | 60.6 | 43.6 | 15.1 |
| Estramustine | 4.1 | 1.5 | 0.5 | 0.4 |
| Cabazitaxel | 0 | 0.6 | 0.4 | 0.4 |
| Abiraterone acetate | 0.7 | 27.7 | 38.0 | 66.9 |
| Enzalutamide | 0 | 0 | 1.6 | 8.7 |
| Sipuleucel-T | 0 | 7.7 | 15.0 | 8.1 |
| Mitoxantrone | 2.4 | 0.4 | 0.2 | 0 |
| Docetaxel, estramustine | 2.1 | 0.9 | 0.7 | 0.2 |
| Total number of LOT1 regimens | 292 | 465 | 566 | 531 |
| **LOT2+ mCRPC drug (%)** |  |  |  |  |
| Docetaxel | 3.7 | 7.6 | 8.1 | 18.2 |
| Estramustine | 3.3 | 1.3 | 0.5 | 0.9 |
| Cabazitaxel | 11.4 | 11.7 | 7.6 | 1.8 |
| Abiraterone acetate | 29.7 | 36.8 | 33.3 | 32.7 |
| Enzalutamide | 8.1 | 15.2 | 27.5 | 28.2 |
| Sipuleucel-T | 1.2 | 1.1 | 1.0 | 0.9 |
| Blank regimen | 27.6 | 20.4 | 15.7 | 8.2 |
| Docetaxel, estramustine | 1.6 | 0.9 | 0.0 | 0.9 |
| Mitoxantrone | 9.8 | 1.1 | 0.7 | 0.9 |
| Total number of LOT2+ regimens | 246 | 446 | 408 | 110 |

Abbreviations: LOT1, first line of treatment; LOT2+, beyond second line of treatment; mCRPC, metastatic castration-resistant prostate cancer.
