# Supplementary material for: Treatment evolution for metastatic castration‐resistant prostate cancer with recent introduction of novel agents: retrospective analysis of real‐world data
Source: Cancer Med. 2015 Dec 29;5(2):182–91. doi: 10.1002/cam4.576 (PMC4735776; doi:10.1002/cam4.576)
Supplement: Supplementary file 5 — Table S4. mCRPC Drug Usage Proportion for LOT1 by Age Group and 1‐Year Cohorts From 2010–2013. [file CAM4-5-182-s005.docx]

**SUPPLEMENTARY TABLE 4.** mCRPC Drug Usage Proportion for LOT1 by Age Group and 1-Year Cohorts From 2010 to 2013

|  | **Commercial Claims Database** | | | | | | | | | | | |
| --- | --- | --- | --- | --- | --- | --- | --- | --- | --- | --- | --- | --- |
|  | **2010** | | | **2011** | | | **2012** | | | **2013** | | |
| **Age group, years** | **44-64** | **65-80** | **>80** | **44-64** | **65-80** | **>80** | **44-64** | **65-80** | **>80** | **44-64** | **65-80** | **>80** |
| **mCRPC drug (%)** |  |  |  |  |  |  |  |  |  |  |  |  |
| Docetaxel | 90.8 | 91.5 | 90.2 | 67.6 | 58.0 | 51.9 | 51.3 | 46.8 | 21.5 | 21.9 | 15.3 | 6.8 |
| Mitoxantrone | 0.8 | 1.5 | 9.8 | 0 | 0.5 | 0 | 0 | 0 | 0.9 | 0 | 0 | 0 |
| Estramustine | 5.9 | 3.8 | 0 | 1.1 | 1.5 | 2.5 | 1.0 | 0.4 | 0 | 0.7 | 0.4 | 0 |
| Abiraterone acetate | 0.8 | 0.8 | 0 | 18.9 | 30.5 | 41.8 | 28.3 | 35.2 | 62.6 | 58.3 | 65.7 | 78.8 |
| Enzalutamide | 0 | 0 | 0 | 0 | 0 | 0 | 0.5 | 2.2 | 1.9 | 5.3 | 10.5 | 9.1 |
| Cabazitaxel | 0 | 0 | 0 | 1.6 | 0 | 0 | 0.5 | 0.4 | 0 | 0 | 0.8 | 0 |
| Sipuleucel-T | 0 | 0 | 0 | 8.1 | 9.0 | 3.8 | 17.3 | 14.2 | 13.1 | 11.9 | 7.3 | 5.3 |
| Docetaxel, estramustine | 1.7 | 2.3 | 0 | 1.6 | 0.5 | 0 | 1.0 | 0.7 | 0 | 0.7 | 0 | 0 |
| Total number of LOT1 regimens | 119 | 130 | 41 | 185 | 200 | 79 | 191 | 267 | 107 | 151 | 248 | 132 |

Abbreviations: LOT1, first line of treatment; mCRPC, metastatic castration-resistant prostate cancer.
