# Supplementary material for: Treatment evolution for metastatic castration‐resistant prostate cancer with recent introduction of novel agents: retrospective analysis of real‐world data
Source: Cancer Med. 2015 Dec 29;5(2):182–91. doi: 10.1002/cam4.576 (PMC4735776; doi:10.1002/cam4.576)
Supplement: Supplementary file 6 — Table S5. mCRPC drug usage proportion for LOT1 by age group from the 2013 commercial claims and EMR Cohortsa. [file CAM4-5-182-s006.docx]

**SUPPLEMENTARY TABLE 5.** mCRPC Drug Usage Proportion for LOT1 by Age Group From the 2013 Commercial Claims and EMR Cohorts^a^

|  | **2013 Cohorts** | | | | | |
| --- | --- | --- | --- | --- | --- | --- |
|  | **Commercial Claims** | | | **EMR** | | |
| **Age group, years** | **44-64** | **65-80** | **>80** | **44-64** | **65-80** | **>80** |
| Docetaxel, n (%) | 33 (22) | 37 (15) | 9 (7) | 16 (28) | 27 (13) | 7 (6) |
| Abiraterone acetate, n (%) | 88 (58) | 163 (66) | 104 (79) | 31 (54) | 129 (63) | 86 (78) |
| Enzalutamide, n (%) | 8 (5) | 26 (11) | 12 (9) | 3 (5) | 24 (12) | 11 (10) |
| Cabazitaxel, n (%) | 0 | 2 (1) | 0 | 0 | 1 (1) | 0 |
| Sipuleucel-T, n (%) | 18 (12) | 18 (7) | 7 (5) | 5 (9) | 16 (8) | 3 (3) |
| Total number of LOT1 regimens | 151 | 248 | 132 | 57 | 204 | 111 |

Abbreviations: LOT1, first line of treatment; EMR, electronic medical record; mCRPC, metastatic castration-resistant prostate cancer.

^a^ mCRPC drug usage proportion was not significantly different between commercial claims and EMR cohorts in the age groups 44-64 (*P* = .76), 65-80 (*P* = .95), >80 (*P* = .8).
