# Supplementary material for: Treatment evolution for metastatic castration‐resistant prostate cancer with recent introduction of novel agents: retrospective analysis of real‐world data
Source: Cancer Med. 2015 Dec 29;5(2):182–91. doi: 10.1002/cam4.576 (PMC4735776; doi:10.1002/cam4.576)
Supplement: Supplementary file 7 — Table S6. Estimated treatment duration (Days) of mCRPC Drugsa in the 2009–2013 Cohorts. [file CAM4-5-182-s007.docx]

**SUPPLEMENTARY TABLE 6.** Estimated Treatment Duration (Days) of mCRPC Drugs^a^ in the 2009 to 2013 Cohorts

|  | **LOT1** | | | | | | **LOT2** | | | | | |
| --- | --- | --- | --- | --- | --- | --- | --- | --- | --- | --- | --- | --- |
|  | **Commercial Claims**  **n = 2138** | | | **EMR^b^**  **n = 1340** | | | **Commercial Claims**  **n = 789** | | | **EMR^b^**  **n = 645** | | |
|  | **n** | **Mean ± SD** | **Median** | **n** | **Mean ± SD** | **Median** | **n** | **Mean ± SD** | **Median** | **n** | **Mean ± SD** | **Median** |
| **Docetaxel** | 995 | 136 ± 147 | 105 | 469 | 165 ± 166 | 112 | 142 | 91 ± 77 | 69 | 140 | 107 ± 87 | 91 |
| **Abiraterone acetate** | 698 | 164 ± 141 | 122 | 335 | 171 ± 133 | 127 | 453 | 167 ± 140 | 118 | 235 | 158 ± 129 | 120 |
| **Enzalutamide** | 55 | 108 ± 81 | 87 | 26 | 112 ± 76 | 98 | 239 | 108 ± 80 | 88 | 106 | 109 ± 65 | 94 |
| **Cabazitaxel** | 5 | 56 ± 18 | 49 | 24 | 183 ± 156 | 140 | 126 | 78 ± 79 | 63 | 165 | 128 ± 106 | 105 |

Abbreviations: EMR, electronic medical record; LOT1, first line of treatment; LOT2, second line of treatment; mCRPC, metastatic castration-resistant prostate cancer; SD, standard deviation.

**^a^** Sipuleucel-T was not included because of the set 3-week treatment course.

^b^ The number of days of supply was assumed to be 30 days for all oral drugs (abiraterone, estramustine, and enzalutamide), as days of supply not contained for prescriptions in EMR data. For oral therapies, patients with single usage of a particular drug were excluded.
